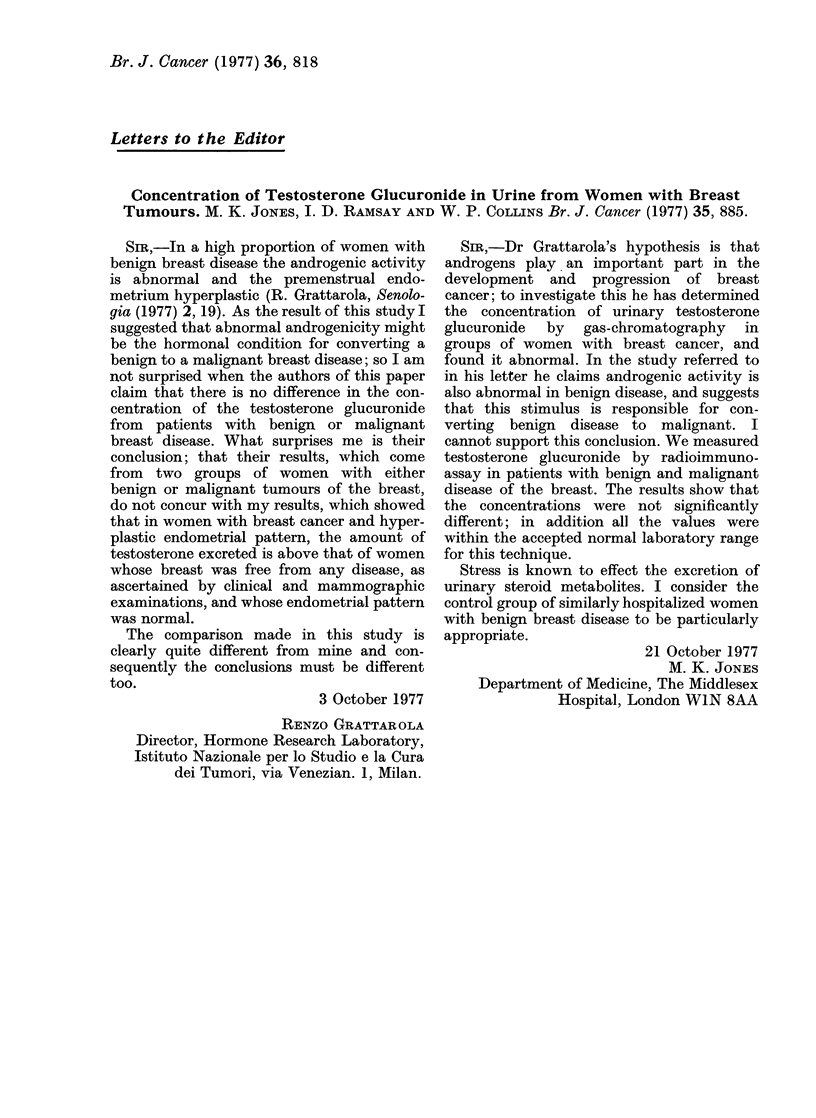# Concentration of Testosterone Glucuronide in Urine from Women with Breast Tumours

**Published:** 1977-12

**Authors:** M. K. Jones


Sir,—Dr Grattarola's hypothesis is that androgens play an important part in the development and progression of breast cancer; to investigate this he has determined the concentration of urinary testosterone glucuronide by gas-chromatography in groups of women with breast cancer, and found it abnormal. In the study referred to in his letter he claims androgenic activity is also abnormal in benign disease, and suggests that this stimulus is responsible for converting benign disease to malignant. I cannot support this conclusion. We measured testosterone glucuronide by radioimmunoassay in patients with benign and malignant disease of the breast. The results show that the concentrations were not significantly different; in addition all the values were within the accepted normal laboratory range for this technique.

Stress is known to effect the excretion of urinary steroid metabolites. I consider the control group of similarly hospitalized women with benign breast disease to be particularly appropriate.

21 October 1977

M. K. Jones
Department of Medicine, The Middlesex
Hospital, London W1N 8AA